# Pancreaticogastrostomy in pure laparoscopic pancreaticoduodenectomy—A novel pancreatic-gastric anastomosis technique -

**DOI:** 10.1186/s12893-015-0061-x

**Published:** 2015-07-02

**Authors:** Masamichi Matsuda, Shusuke Haruta, Hisashi Shinohara, Kazunari Sasaki, Goro Watanabe

**Affiliations:** Department of Surgery, Toranomon Hospital, 2-2-2 Toranomon Minato-ku, Tokyo, 105-8470 Japan

**Keywords:** Laparoscopy, Pancreaticoduodenectomy, Pancreatic surgery, Laparoscopic pancreaticoduodenectomy, Pancreaticogastrostomy, Pancreatic-enteric anastomosis

## Abstract

**Background:**

Although many surgical procedures are now routinely performed laparoscopically, pure laparoscopic pancreaticoduodenectomy (LPD) is not commonly performed because of the technical difficulty of pancreatic resection and the associated reconstruction procedures. Several pancreatic-enteric anastomosis techniques for LPD have been reported, but most are adaptations of open procedures. To accomplish pure LPD, we consider it necessary to establish new pancreatic-enteric anastomosis techniques that are specifically developed for LPD and are safe and feasible to perform.

**Results:**

One patient developed a postoperative pancreatic fistula (International Study Group of Pancreatic Fistula criteria, grade B) and subsequent postoperative delayed gastric emptying (International Study Group of Pancreatic Surgery criteria, grade C). No other major complications occurred. We developed a novel pancreatic-gastric anastomosis technique that enabled us to safely perform pure LPD. The main pancreatic duct was stented with a 4-Fr polyvinyl catheter during pancreatic resection. A small hole was created in the posterior wall of the stomach and was bluntly dilated. A 5-cm incision was made in the anterior stomach, and the pancreatic drainage tube was passed into the stomach through the hole in the posterior wall. The remnant pancreas was pulled into the stomach, and was easily positioned and secured in place with only four to six sutures between the pancreatic capsule and the gastric mucosa. We used this technique to perform pure LPD in five patients between December 2012 and July 2013.

**Conclusions:**

Our new technique is technically easy and provides secure fixation between the gastric wall and the pancreas. This technique does not require main pancreatic duct dilatation, and the risk of intra-abdominal abscess formation due to postoperative pancreatic fistula may be minimized. Although this technique requires further investigation as it may increase the risk of delayed gastric emptying, it may be a useful method of performing pancreaticogastrostomy in pure LPD.

**Trial registration:**

ISRCTN16761283. Registered 16 January 2015.

**Electronic supplementary material:**

The online version of this article (doi:10.1186/s12893-015-0061-x) contains supplementary material, which is available to authorized users.

## Background

Although many surgical procedures are now routinely performed laparoscopically, pure laparoscopic pancreaticoduodenectomy (LPD) is not widely performed because of the technical difficulty of pancreatic resection and the complexity of reconstruction procedures. As postoperative pancreatic anastomotic leakage carries an increased risk of intra-abdominal hemorrhage and a high mortality rate [1, 2], some surgeons avoid intracorporeal reconstruction, and use a hybrid laparoscopic-open approach to increase the safety and the feasibility of pancreatic anastomosis [3, 4]. Although a hybrid approach may reduce operative risk, it also results in loss of the potential advantages of minimally invasive surgery. Several reports have described techniques for laparoscopic pancreaticojejunostomy (PJ), but most are adaptations of open procedures [5–10]. Just as in open surgery, LPD carries an increased risk of postoperative pancreatic fistula formation in patients with soft pancreatic texture or a small pancreatic duct. This increased risk may be attributed to the technical difficulty of performing the traditional duct-to-mucosa anastomosis in the pancreatic-enteric reconstruction. Magnification laparoscopy can be useful for this duct-to-mucosa anastomosis, but the restricted range of motion of laparoscopic forceps sometimes makes it difficult to perform the anastomosis. There have been an increasing number of robotic PD which may facilitate execution of complex reconstruction, but robotic PD is feasible only for highly selected patients [11]. We therefore consider it necessary to establish novel pancreatic-enteric anastomosis techniques that are simple, feasible to perform, provide secure fixation between the enteric wall and the pancreas, and are specifically developed for LPD. We describe herein our novel pancreatic-gastric anastomosis technique (PG) in pure LPD.

## Methods

From December 2012 to July 2013, we used our technique in five patients. Patients were eligible for this procedure if they were non-obese with no previous upper abdominal surgery. Three patients had intraductal papillary mucinous neoplasm, one had carcinoma of the papilla of Vater, and one had solid pseudopapillary neoplasm. Before surgery, the tumors were fully evaluated by abdominal computed tomography, magnetic resonance imaging, and endoscopic ultrasonography. All the tumors were restricted to the pancreatic head or periampullary region. The patients were three males and two females with a median age of 64 years (range, 47–76 years) and a median body mass index of 22.2 kg/m^2^ (range, 17.4–25.5 kg/m^2^). The patients were all East Asian and lived in the eastern part of Japan. The advantages, disadvantages, and potential risks of the surgical procedure were explained to patients and informed consent was obtained. Data recording and evaluation was approved by the ethics committee of Toranomon Hospital and was in accordance with the Declaration of Helsinki.

### Technique

Surgery was performed under general anesthesia with the patient in the supine position with the legs apart. The first 12-mm umbilical trocar was inserted for an electrolaparoscope (LTF-VH, Olympus Medical Systems, Tokyo, Japan) using a mini-laparotomy technique, and a pneumoperitoneum was established with a CO_2_ pressure of 10 mmHg. Three 12-mm trocars (left subcostal and bilateral supraumbilical pararectal) and one 5-mm trocar (right subcostal) were placed in the abdominal wall. The surgeon stood on the right side of the patient during reconstruction. After mobilization of the head of the pancreas, a tunnel was formed between the posterior aspect of the neck of the pancreas and the superior mesenteric and portal veins. If possible, the tunnel was extended 2–3 cm towards the body of the pancreas in preparation for easy reconstruction. The body of the pancreas was then slowly and gently dissected using laparoscopic coagulation shears (SonoSurg™, Olympus Medical Systems). The main pancreatic duct was identified, and was cut across half its width with scissors and then stented with a 4-Fr polyvinyl catheter (MD-41513 pancreatic duct tube, 65 cm; Sumitomo Bakelite, Tokyo, Japan). The tube was firmly attached to the distal pancreas in two places with 3–0 absorbable sutures (Vicril™ 3–0, Ethicon).

### Reconstruction

**Fig. 1:**
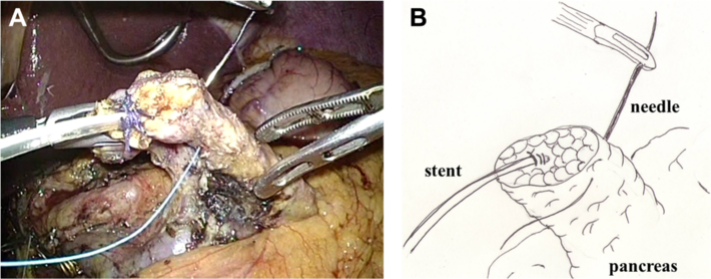
Two anchoring sutures were placed in the remnant pancreas, 2 cm distal to the transection plane. The main pancreatic duct was already stented with a 4-Fr polyvinyl catheter. A: photo, B: illustration

**Fig. 2:**
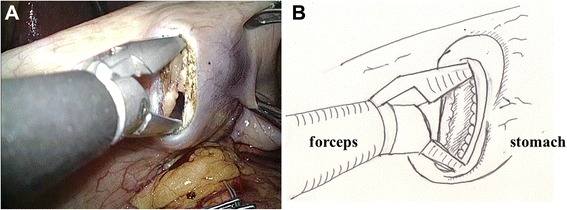
A small hole was made in the gastric serosa at the planned anastomotic site, and was bluntly dilated with forceps. A: photo, B: illustration

**Fig. 3:**
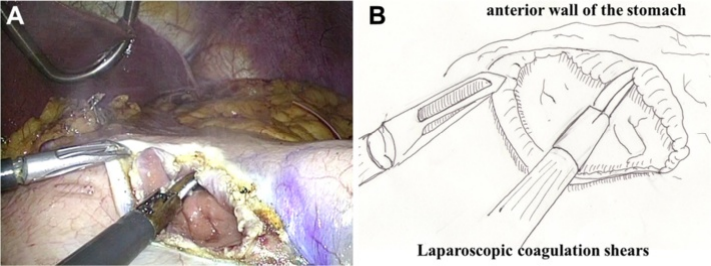
A 5-cm vertical incision was made in the anterior wall of the stomach just ventral to the planned anastomotic site, using laparoscopic coagulation shears. A: photo, B: illustration

**Fig. 4:**
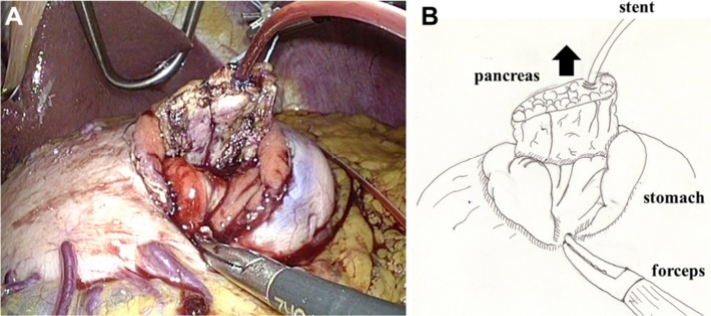
The two anchoring sutures and the stenting tube were passed through the hole, and the remnant pancreas was then pulled into the stomach and fixed in place. A: photo, B: illustration

**Fig. 5:**
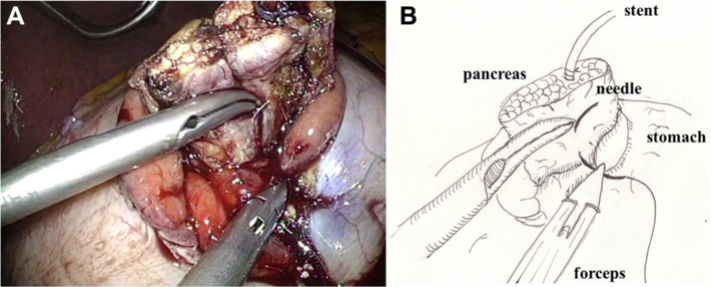
After pulling the remnant pancreas 2–3 cm into the stomach, four to six interrupted sutures were placed between the pancreatic capsule and the gastric mucosa. A: photo, B: illustration

**Fig. 6:**
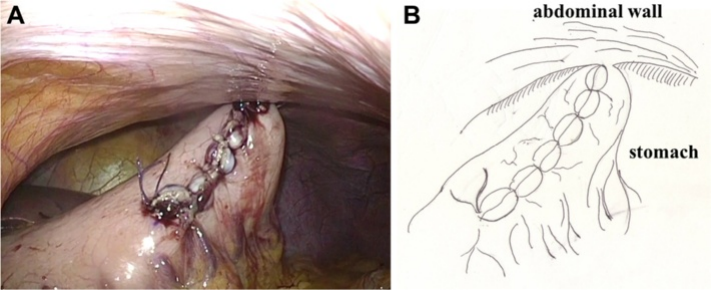
The stenting tube was passed through the abdominal wall to form a gastrostomy. A: photo, B: illustration

After excision of the proximal portion of the pancreas, the specimen was removed via the umbilical trocar site, which was extended to 3 cm. The distal portion of the remnant pancreas was dissected from the splenic artery, splenic vein, and connecting tissues with laparoscopic coagulation shears, for up to 3 cm beyond the transection plane, in preparation for invagination into the stomach. Two anchoring sutures (Ti-Cron™ 3–0, Covidien) were placed in the remnant pancreas, 2 cm distal to the transection plane (Fig. 1 and Additional file 1). After deciding the site of the anastomosis (usually the posterior wall of the lower body of the stomach), a small hole was made in the gastric serosa at the planned anastomotic site by electrocautery, and the hole was bluntly dilated with forceps (Fig. 2 and Additional file 2). A 5-cm vertical incision was then made in the anterior wall of the stomach just ventral to the planned anastomotic site with laparoscopic coagulation shears (Fig. 3 and Additional file 3). The two anchoring sutures and the stenting tube were passed through the hole at the anastomotic site and pulled into the stomach using forceps introduced through the gastric incision. The remnant pancreas was then pulled into the stomach through the hole at the anastomotic site and fixed in place with the anchoring sutures, taking care not to injure the pancreas (Fig. 4 and Additional file 4). After pulling the remnant pancreas 2–3 cm into the stomach, four to six interrupted sutures (Vicril™ 3–0, Ethicon) were placed between the pancreatic capsule and the gastric mucosa (Fig. 5 and Additional file 5). The stenting tube was passed through the incision in the anterior wall of the stomach, and the incision was closed with a continuous absorbable suture (PDS™ 4–0, Ethicon). The stenting tube was then passed through the abdominal wall (usually left subcostal) to form a gastrostomy (Fig. 6). Fibrin glue was placed around the PG site for protection. A prophylactic drainage tube (Multi-Channel™ Drainage Set 6.5 mm, Covidien) was placed at the pancreatic anastomosis.

## Results

All the procedures were performed by the same surgeon (Table 1). The pancreatic texture was soft in all patients. The median estimated blood loss was 100 ml (range, 0–400 ml) and the median operative time was 492 min (range, 435–739 min). The median hospital stay was 35 days (range, 19–57 days). The external drainage tube was left in place for a median of 20 days after surgery (range, 18–22 days), and the drainage tube at the anastomotic site was left in place for a median of 24 days after surgery (range, 17–41 days). One patient developed a postoperative pancreatic fistula of International Study Group of Pancreatic Fistula (ISGPF) grade B [12] on postoperative day 7, and subsequently developed postoperative delayed gastric emptying of International Study Group of Pancreatic Surgery (ISGPS) grade C [13]. Both these complications resolved with conservative management. No major complications occurred in the other patients, and the postoperative follow-up period was uneventful (range, 7.7–15.5 months). All resection margins were tumor-free on frozen section examination.

**Table 1:** Patients demographics and surgical outcomes

Case	Diagnosis	ope time (min)	blood loss (ml)	stenting tube	PF (ISGPF)	DGE (ISGPS)	Length of stay (days)
1	Ampullary ca	492	0	internal	B	C	50
2	IPMN	739	350	external	0	A	57
3	IPMN	599	400	external	0	A	35
4	IPMN	435	100	external	0	A	29
5	SPN	450	100	external	0	0	19

*Ampullary ca* carcinoma of the ampulla of Vater, *IPMN* intraductal papillary mucinos neoplasm, *SPN* solid pseudo-papillary neoplasm, *PF* pacreatic fistula, *DGE* delayed gastric emptying

## Discussion

The incidence of postoperative pancreatic fistula ranges from 2 to more than 20 % after open pancreaticoduodenectomy [13], and from 1.8 to 20 % after LPD [6, 7, 9, 10]. It is important to achieve a good pancreatic-enteric anastomosis, because a postoperative pancreatic fistula may lead to major complications, prolonged hospital stay, and mortality [1, 2]. Minilaparotomy has been advised to ensure safe anastomosis. Although a hybrid laparoscopic-open technique may reduce operative risk, it also results in loss of the potential advantages of minimally invasive surgery. We developed a new PG technique to enable safe reconstruction in pure LPD.

Although PG has been considered an acceptable method of reconstruction after pancreaticoduodenectomy over the past 50 years, there is still controversy regarding the relative superiority of PG versus PJ in terms of outcomes. Wellner et al. reported that PG was superior to PJ in terms of postoperative pancreatic fistula formation judged according to the ISGPF criteria [14]. Also the recent meta-analysis done on PJ vs PG after PD revealed that PG seems to be superior to PJ in reducing the incidence of pancreatic fistula formation and intra-abdominal fluid collection [15–17].

The standard pancreatic-enteric anastomosis performed during LPD is PJ. Only one published case report has described reconstruction with PG in LPD [18]. In that case, the remnant pancreas was invaginated into the stomach and was fixed in place with two continuous purse-string sutures around the incision in the gastric wall using self-retaining monofilament sutures (V-Loc 180 3–0, Covidien). Our technique is relatively simpler to perform. We created a small hole in the posterior wall of the stomach and dilated it bluntly. The remnant pancreas was then pulled into the stomach, and easily positioned so that only a few sutures were required between the pancreatic capsule and gastric mucosa to hold it in place.

In LPD, reconstruction is usually performed by end-to-side PJ with duct-to-mucosa anastomosis [5–7]. Just as in open surgery, LPD carries an increased risk of pancreatic fistula formation in patients with a small pancreatic duct. This increased risk may be attributed to the technical difficulty of performing the duct-to-mucosa anastomotic portion of the pancreatic-enteric reconstruction. In such patients, magnification laparoscopy can be useful for performing duct-to-mucosa anastomosis, but the restricted range of motion of laparoscopic forceps sometimes makes this anastomosis difficult. Our technique does not require duct-to-mucosa anastomosis, and it can be easily used in patients with a small pancreatic duct.

Our technique may also reduce the risk of intra-abdominal abscess formation due to minor leakage of pancreatic juice from the injured pancreatic capsule, because the sutures between the pancreas and the gastric wall are placed inside the stomach. As damage to the pancreatic capsule outside the stomach can be avoided, this technique may be safe in patients with a soft pancreatic texture.

One patient in our series developed a postoperative pancreatic fistula (ISGPF grade B). In this patient, only two sutures were placed between the pancreatic capsule and the gastric mucosa, which was probably inadequate and may have contributed to fistula formation. To reduce the risk of postoperative pancreatic fistula after PG, we suggest placement of a sufficient number of sutures between the pancreatic capsule and the gastric mucosa.

This patient also received only a short internal plastic stent across the PG site. A meta-analysis of randomized clinical trials found that placement of a stent in the pancreatic duct did not reduce the incidence of postoperative pancreatic fistula. However, subgroup analysis found that use of an external stent significantly reduced the incidence of postoperative pancreatic fistula [19]. Other randomized clinical trials found that external duct stenting after pancreaticoduodenectomy reduced the risk of clinically relevant postoperative pancreatic fistula formation [20, 21]. The majority of the selected patients of these studies used PJ for reconstruction, and subgroup analysis for external duct stenting in PG was not reported. Placement of an external stent across the PG anastomosis is not necessarily an essential part of PG, but could be used adjunct to reduce the risk of pancreatic fistula formation.

One of the disadvantages of our technique is that it may result in delayed gastric emptying, which is one of the most common postoperative complications after pancreatic surgery, occurring in 19–57 % of patients [13]. In patients with PG, gastric peristalsis is disturbed because the posterior wall of the stomach is held in place by the PG anastomosis. Additionally, incision of the anterior wall of the stomach increases the risk of delayed gastric emptying [22]. In our technique, the anterior wall of the stomach is incised, sutured, and attached to the abdominal wall by the gastrostomy, which may cause delayed gastric emptying.

The long-term oncologic and surgical outcomes after use of our procedure should be investigated, and future research should investigate whether LPD provides any significant advantages over other methods of performing pancreaticoduodenectomy. It is difficult to draw any sound conclusions about the safety or limitations of our technique with so little information about patient selection, but we consider our technique a relatively easy method for reconstruction in pure LPD. Our technique may also provide an alternative reconstruction method for use in a hybrid procedure. As reconstruction with PG in LPD is still a new technique, further clinical evaluation to compare outcomes between the use of PG and PJ in LPD is warranted.

## Conclusions

We present a novel pancreatic-gastric anastomosis technique specifically developed for LPD. Our new technique is technically easy and provides excellent fixation between the gastric wall and pancreas. Main pancreatic duct dilatation is not required, and the risk of intra-abdominal abscess formation is minimized. Although further clinical evaluation is required, this technique is immediately clinically applicable and may serve as the basis for additional research.

## Additional files

Additional file 1:
**Two anchoring sutures were placed in the remnant pancreas, 2 cm distal to the transection plane.** The main pancreatic duct was already stented with a 4-Fr polyvinyl catheter. Additional file 2: A small hole was made in the gastric serosa at the planned anastomotic site, and was bluntly dilated with forceps. Additional file 3: A 5-cm vertical incision was made in the anterior wall of the stomach just ventral to the planned anastomotic site, using laparoscopic coagulation shears. Additional file 4: The two anchoring sutures and the stenting tube were passed through the hole, and the remnant pancreas was then pulled into the stomach and fixed in place. Additional file 5: After pulling the remnant pancreas 2–3 cm into the stomach, four to six interrupted sutures were placed between the pancreatic capsule and the gastric mucosa.

## Abbreviations

LPD: Laparoscopic pancreaticoduodenectomy
PJ: Pancreaticojejunostomy
PG: Pancreaticogastrostomy
ISGPF: International Study Group of Pancreatic Fistula
ISGPS: International Study Group of Pancreatic Surgery
